# Nomenclatural Synopsis, Revised Distribution and Conservation Status of *Ranunculus gracilis* (Ranunculaceae) in Italy

**DOI:** 10.3390/plants11223094

**Published:** 2022-11-14

**Authors:** Fabrizio Bartolucci, Enzo De Santis, Fabio Conti

**Affiliations:** 1 Centro Ricerche Floristiche dell’Appennino, Parco Nazionale del Gran Sasso e Monti della Laga—Scuola di Bioscienze e Medicina Veterinaria, Università di Camerino, San Colombo, Barisciano, 67021 L’Aquila, Italy; 2 Independent Researcher, 00189 Roma, Italy

**Keywords:** endemism, floristic research, herbaria, lectotype, Mediterranean flora, nomenclature, taxonomy

## Abstract

*Ranuculus gracilis* is endemic to the SE Euro-Mediterranean area and its presence in Italy is controversial. Based on analysis of the relevant literature, field surveys and examination of herbarium specimens, a revised distribution of this species in Italy is presented and its conservation status is assessed. *Ranunculus agerii*, described by Antonio Bertoloni from Bologna (Emilia-Romagna, Northern Italy), and *R. schowii,* described by Vincenzo Tineo from Vittoria and Terranova (Sicily), usually regarded as synonyms of *R. gracilis*, are here lectotypified and their taxonomic status discussed. Thanks to our study, the presence of *R. gracilis* in Italy is confirmed and, now, it is reported in a national conservation framework.

## 1. Introduction

*Ranunculus* L. is the largest genus in the family *Ranunculaceae* Juss., with a cosmopolitan distribution, consisting of about 1200 species (including also ca. 600 agamospecies) [1,2]. Based on morphological and molecular data, the genus *Ranunculus* was divided into two subgenera (subg. *Auricomus* and subg. *Ranunculus*) and 17 sections [2]. In Italy, the genus *Ranunculus* comprises 112 taxa (species, subspecies and agamospecies), of which 33 are endemic (four are extinct) and one is an alien species [3,4,5].

*Ranuculus gracilis* was described in 1814 by Edward Daniel Clarke from the East Aegean island of Kos (Greece) [6] and belongs to *R.* subg. *Ranunculus* sect. *Ranunculastrum* DC. [2]. This species is endemic to the SE Euro-Mediterranean area and it is distributed in Italy, the Balkan Peninsula, Turkey and Georgia [7,8]. Contrary to what is reported by Euro+Med Plantbase [7] and POWO [8], the presence of *R. gracilis* in Italy is controversial, i.e., [4,9]. According to the latest Italian Flora [9], it is present in a few localities in Sicily and Calabria, no longer recorded in Emilia-Romagna and cultivated in Umbria. On the contrary, following the Italian Checklist [4], it is recorded by mistake in Piemonte, Emilia-Romagna, Umbria, Sicily and doubtfully occurring in Puglia. Two species, described from Italy, are usually regarded as synonyms of *R. gracilis* (e.g., [9,10,11,12]): *R. agerii* Bertol. and *R. schowii* Tineo (with doubt). *Ranunculus agerii* was described in 1819 by Antonio Bertoloni from the areas around Bologna (Emilia-Romagna, northern Apennines, Italy) [13] and it is currently regarded as a synonym of *R. gracilis* (i.e., [7,8,9,12,14,15,16,17]). *Ranunculus schowii* was described by Vincenzo Tineo in Gussone [18] from Vittoria and Terranova (Sicily) and regarded as a dubious synonym of *R. gracilis* or *R. agerii* by some authors (i.e., [10,11,12]) or as a synonym of *R. monspeliacus* L. subsp. *monspeliacus* (i.e., [8]). The purpose of this study is to critically review the presence of *R. gracilis* in Italy and to understand the taxonomic identity of *R. agerii* and *R. schowii*, two names that turned out to be, to the best of our knowledge, not yet typified.

The present contribution is part of an ongoing project promoted by the Italian Botanical Society, aimed at recognizing and typifying all the taxa described from Italy, in order to increase their systematic knowledge and promote further studies [19,20,21,22].

## 2. Materials and Methods

This study is based on an extensive analysis of relevant literature, field surveys and examination of herbarium specimens (including the original material) preserved in APP, BOLO, BR, CAT, CHE, CLF, DR, E, FI, G, GOET, JE, MW, NAP, P, PI, PAL, W and WU (the acronyms follow [23]). We performed a survey for original material for the name *R. agerii* at BOLO where Bertoloni’s main collection is housed and at FI, G, NAP and PAL to trace the original material of the name *R. schowii* (see [24,25]). The original material for the name *R. gracilis* was searched at A, BM, CAN, CGE, ECON, GH, FI, K and SWN. The type designations herein follow the Shenzhen Code ([26], hereafter ICN).

The revised Italian distribution of *R. gracilis* is based on examination of herbarium specimens. The distribution data and the occurrence status are given for the Italian administrative regions according to Bartolucci et al. [4].

## 3. Results

### 3.1. The Long and Controversial History of R. gracilis in Italy

Over the years, the presence of *R. gracilis* in Italy has been controversial due to the confusion in its identification and to the unclear taxonomic relationships with some currently not accepted species [7,8], such as *R. agerii* Bertol., *R. schowii* Tin. and *R. chaerophyllos* L.

The first herbarium samples collected in Italy that can be referred with certainty to *R. gracilis* date back to the 16th century. There are two specimens without collection locality preserved in the “*En Tibi*” herbarium, made by Francesco Petrollini in Bologna around 1558, kept in Leiden (an image of the specimen is available at https://data.biodiversitydata.nl/naturalis/specimen/L.2110949 (accessed on 10 September 2022)) and in the controversial “Cibo” herbarium kept in Rome in Biblioteca Angelica, recently also attributed to Petrollini [27,28]. According to Stefanaki et al. [28], it is evident that many specimens in the “*En Tibi*” herbarium were collected in the area of Bologna, where Petrollini had his place of residence.

Towards the end of 1500, this plant was also collected by Nicolas Ager (or Agerius) in the Bologna hills. He sent samples to Jean Bauhin, who gave a brief description of this plant in the *Phytopinax* published by Caspar Bauhin as “*Ranunculus racemosa radice Io. Bauhini* … *Reperitur in montibus Bononiensibus*” [29]. Later, it was reported by the Bauhin brothers as “*Ranunculus grumosa radice folio ranunculi bulbosi* … *Hic Ranunculus agris Bononiensibus familiaribus est, & à D. Agerio collectus*” in the *Prodromos theatri botanici* [30], “*Ranunculus grumosa radice folio ranunculi bulbosi*” in the *Pinax theatri botanici* [31], “*Ranunculus racemosa radice* … *Agerio siccam dedit pro Ranunculo Chelidoniae radice*” in the *Historia plantarum universalis* [32] and by Parkinson [33] as “*Ranunculus grumosa radice Bononiensis*”. Later, Linnaeus [34], mistakenly included the polynomial published by C. Bauhin in the *Prodromus* [30] and in the *Pinax* [31] in *R. chaerophyllos* L. Antonio Bertoloni was the first to accurately describe [13] the plant from the areas around Bologna (Emilia-Romagna) as “*Ranoncolo Bolognese*”, dedicating it to Nicolas Ager, with the name *R. agerii*. After the description, *R. agerii* was treated as synonym of *R. chaerophyllos* L. by Arcangeli [35] and Cesati et al. [36], a name of uncertain application [37]. Later, Fiori et al. [10] and Fiori [11] re-evaluated *R. agerii* as a good species, recording it not only for its *locus classicus*, but also for Sicily and quoting *R. gracilis* and, with doubt, *R. schowii* Tineo, only in [11] as synonyms. Pons [38] recorded *R. agerii* (syn. *R. gracilis*) for several localities around Bologna in Emilia-Romagna and for Catania in Sicily. Tutin [14] and Tutin and Akeroyd [17] in Flora Europaea quoted *R. gracilis* (syn. *R. agerii*) for Italy and Sicily. Zangheri [39] reported *R. gracilis* (syn. *R. agerii*) for Sicily and as naturalized in Northern and Central Italy. Pignatti [15] reported *R. gracilis* (syn. *R. agerii*) in Sicily and no longer recorded for Emilia-Romagna. Greuter et al. [40] in the Med-Checklist quoted *R. gracilis* (syn. *R. agerii*) for Sicily and as doubtfully native in Italy. Jalas and Suominen [16] quoted *R. gracilis* (syn. *R. agerii*) for Calabria and Sicily and with doubt in Emilia-Romagna. Peruzzi and Passalacqua [41] reported *R. gracilis* for the Balkan Peninsula, Turkey, and Crete, while, the Italian records of Calabria and Sicily, should be referred to *R. monspeliacus* L. subsp. *aspromontanus* (Huter, Porta & Rigo) Peruzzi & N.G.Passal. Conti et al. [42] reported *R. gracilis* without synonyms as doubtfully occurring in Italy in Piemonte, Emilia-Romagna and Sicily. In the same year, Scoppola and Spampinato [43] recorded *R. gracilis* (syn. *R. agerii*) for Sicily and as indicated by mistake in Emilia-Romagna. Later, Conti et al. [44] reported *R. gracilis* as indicated by mistake in Italy, updating the occurrence status in Conti et al. [42]. Recently, Pignatti et al. [9] quoted *R. gracilis* (syn. *R. agerii*) as present in a few localities in Sicily, Calabria, no longer recorded in Emilia-Romagna and cultivated in Umbria. On the contrary, according to Bartolucci et al. [4], in the updated checklist of Italian vascular Flora, the species was recorded by mistake in Piemonte, Emilia-Romagna, Umbria, Sicily and as doubtfully occurring in Puglia. Recently, Guarino and La Rosa [45] in the Digital Flora of Italy included in the 4^th^ volume of Flora of Italy [46], recorded *R. gracilis* for Calabria, alien in Umbria and as doubtfully occurring in Sicily and Emilia-Romagna.

### 3.2. Typification of the Names

#### 3.2.1. *Ranunculus agerii* Bertol., Opusc. Sci. 3: 182. 1819

Protologue citation: [Italy, Emilia-Romagna] “Copiosae provenit Bononiae in campis collinis di Monte Donato prope fodinas gypsi inter sata. Floret Aprili. Perenn.”.

**Figure 1:**
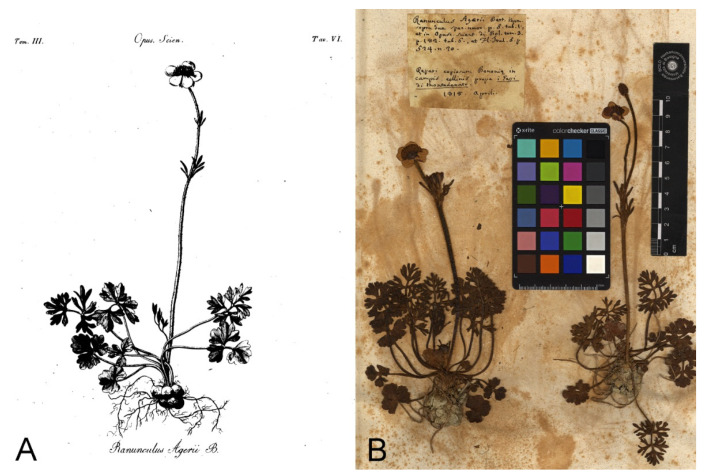
*Ranunculus agerii*: (**A**) illustration cited in the protologue by Bertoloni as “Tab VI”; (**B**) lectotype of the name *R. agerii* kept in BOLO (reproduced with permission of the *Herbarium Bononiensis*, Alma Mater Studiorum University of Bologna, Italy).

Lectotype (designated here): [Italy] Reperi copiosum Bononia in/campis collinis prope i Gessi di Montedonato/1818. Aprili. (BOLO [digital photo!], Figure 1B).

Nomenclatural notes: Antonio Bertoloni [13] described *R. agerii,* providing a detailed description, quoting a precise collection locality and citing an illustration “Tab VI”. In BOLO, where Bertoloni’s main collection is housed, we traced only one herbarium sample with two mounted individuals collected in 1818 on Monte Donato (Figure 1) that can be considered as part of the original material, as well as the illustration “Tab VI” cited in the protologue (Figure 1) (Art. 9.4 of the ICN). The herbarium sample kept in BOLO is complete, well conserved and agrees with the protologue and is selected here as a lectotype for the name *R. agerii*.

Taxonomic notes: based on the original material studied, *R. agerii* should be regarded as a heterotypic synonym of *R. gracilis*.

#### 3.2.2. *Ranunculus gracilis* E.D.Clarke, Travels Eur. Asia and Africa 3 part 2(2): 336. 1814

Protologue citation: [Greece] “island of Cos [Kos]”.

Type: not traced.

Nomenclatural notes: According to Miller [47], Clarke’s herbarium was included in the Herbarium of A.B Lambert. Later, Clarke’s specimens collected in Greece and Asia Minor were bought by G.S. Gibson and subsequently acquired by the herbarium BM. We searched for the original material in BM and in other herbaria where parts of the Lambert and Gibson collections are kept (e.g., CAN, CGE, BM, FI, FH, K and SWN), without finding original material.

#### 3.2.3. *Ranunculus schowii* Tineo, in Gussone Fl. Sicul. Syn. 2(2): 889. 1845. [1 January–September 1845]

Protologue citation: [Italy, Sicily] “In arvis arenosis: fra Vittoria e Terranova (Tin.)”.

**Figure 2:**
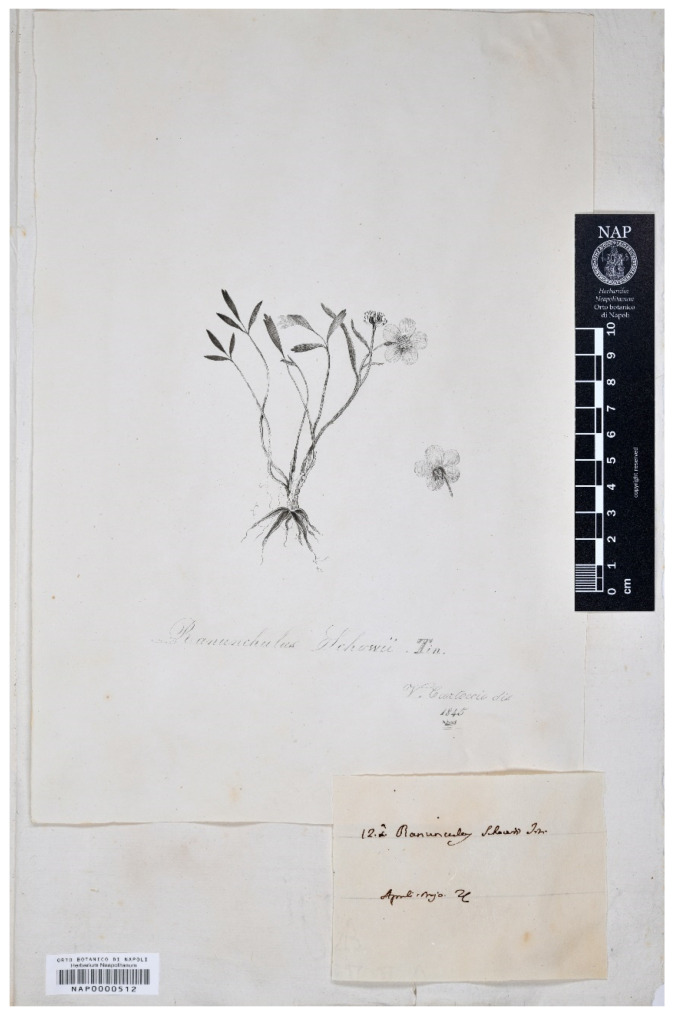
Lectotype of the name *R. schowii* kept in NAP (reproduced with permission of the *Herbarium Neapolitanum*, University of Naples Federico II, Italy).

Lectotype (designated here): [Italy, Sicily [Illustration: *Ranunculus schowii* Tin./V. Cartoccio dis./1845 (NAP barcode NAP0000512 [digital photo!], Figure 2).

Nomenclatural notes: Vincenzo Tineo in Gussone [18] described *R. schowii,* providing a detailed description, quoting a precise collection locality and citing an unpublished illustration “Tin. ined.”. In order to trace the original material, we checked the PAL herbarium, where Tineo’s main collection is housed. We also searched in FI, G, NAP and P, where duplicates by Tineo’s collections are kept. Lojacono Pojero [48] wrote that he saw the only authentic specimen of *R. schowii* in H. Pan. (i.e., *Herbarium Panormitanum*), today PAL. In the *Herbarium Mediterraneum Panormitanum* (PAL), this sample is no longer present (G. Domina, pers. comm.). In NAP (ex-Herbarium Gussone Sicilia), where duplicates by Tineo’s specimens are usually hosted, we did not trace any samples but only the unpublished illustration (“*Ranunculus schowii* Tin./V. Cartoccio dis./1845”; NAP barcode NAP0000512) cited in the protologue. The illustration is also labelled with a representation label [49] “12a. *Ranunculus schowii* Tin./Aprile, Majo”, where “12a” is a reference to the position of the species within the genus in Gussone’s Synopsis. We were not able to trace original material in FI, G and P. The unpublished illustration in NAP (NAP0000512) is the only element belonging to the original material (Art. 9.4 of the ICN), agrees with the protologue and is here designated as the lectotype for the name *R. schowii* (Figure 2).

Taxonomic notes: based on the protologue and the lectotype, *R. schowii* seems to have unique characteristics, only marginally close to particular forms of *R. isthmicus* Boiss. The individual depicted in the illustration (lectotype) shows fusiform root tubers, basal leaves tripartite with entire or lobed (only at the apex) segments and deflexed sepals at flowering. Further studies to assess the morphological variability in this species and to clarify its taxonomic status will be needed. In the case of synonymy of *R. isthmicus* Boiss. (published in 1846, [50]) and *R. schowii* (published in 1845, [18]), the latter would have priority and it should be advisable to proceed with a formal conservation proposal for the name *R. isthmicus*.

### 3.3. Taxonomic Treatment

*Ranunculus gracilis* E.D.Clarke, Travels Eur. Asia and Africa 3 part 2(2): 336. 1814

= *Ranunculus agerii* Bertol., Opusc. Sci. 3: 182. 1819

Lectotype (designate here): [Italy] Reperi copiosum Bononia in/campis collinis prope i Gessi di Montedonato/1818. Aprili. (BOLO [digital photo!], Figure 1B)

= *Ranunculus granulatus* Griseb., Spic. Fl. Rumel. 1: 306. 1843

Lectotype (designated by Strid [51] (p. 306)): [Turkey] *In m. Bulgurlu, Grisebach 24* (GOET barcode GOET009797 [digital photo!]).

= *Ranunculus peloponnesiacus* Boiss., Diagn. Pl. Orient. 1: 63. 1843

Lectotype (designated by Strid [52] (p. 49)): [Greece] *colles elati Argolidis et Arkadiae, Apr. 1842, Boissier s.n.* (P, isolectotype MW barcode MW0592427 [digital photo !]; an image of the isolectotyoe is available at https://plant.depo.msu.ru/public/scan.jpg?pcode=MW0592427 (accessed on 10 September 2022)).

–*Ranunculus chaerophyllos* auct. fl. ital. p.p., non L.

Description (based on [52,53] and personal observations on the studied material): perennial, softly hirsute, 5–40 cm. Root tubers broadly ovate, mixed with long filiform roots. Stems erect, 1 to 3-flowered, appressed-pubescent in upper part, glabrous in the lower part. Basal leaves glabrous to subglabrous, long-petiolate, with the petiole sparsely pilose, dilated into a scarious sheath below; often heteromorphic, outer ones 3-lobed or 3-partite with cuneate-spreading segments divided into obtuse, ovate or oblong lobes, the inner 3-partite to or near base, with lobes variously dissected into obtuse segments. Cauline leaves few and reduced, subsessile, with linear segments. Sepals 6–8 mm, broadly lanceolate, strongly deflexed, sparsely pilose to subglabrous. Petals 6–14 mm, obovate, obtuse, bright yellow. Receptacle glabrous. Achenes numerous, in a dense, narrowly ellipsoid to ovate head (6)8–10 × 4–5 mm Achenes ovate-oblong, wingless, compressed, smooth, c. 1.5–2 mm, attenuate into a straight or somewhat hooked beak 0.5–1.0 mm.

**Figure 3:**
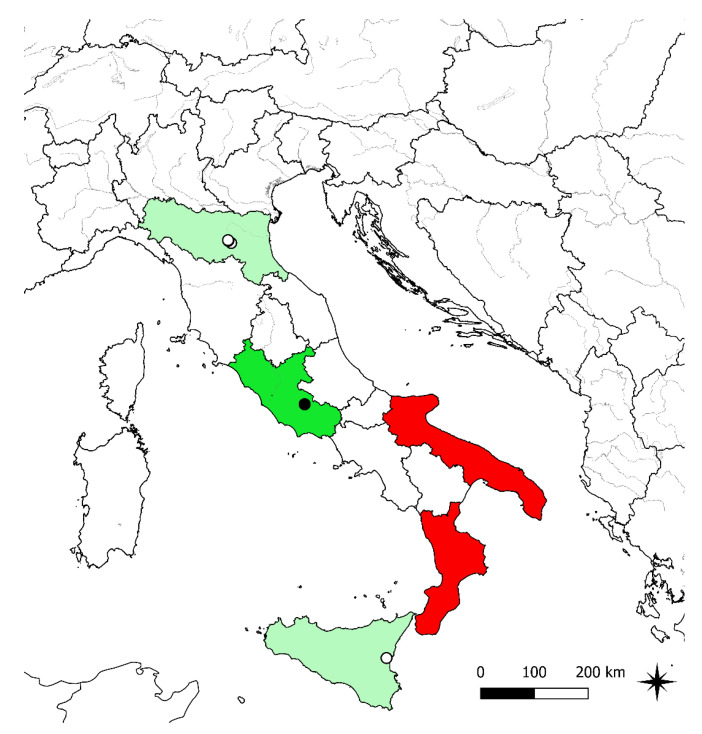
Distribution map of *Ranunculus gracilis* in Italy according to the herbarium material studied and field investigations: black symbols indicate the population currently present based on field investigations and empty symbols refer to the old herbarium specimens seen. Green background: occurring; pale green background: no longer recorded (reliable historical record); red background: recorded by mistake.

**Figure 4:**
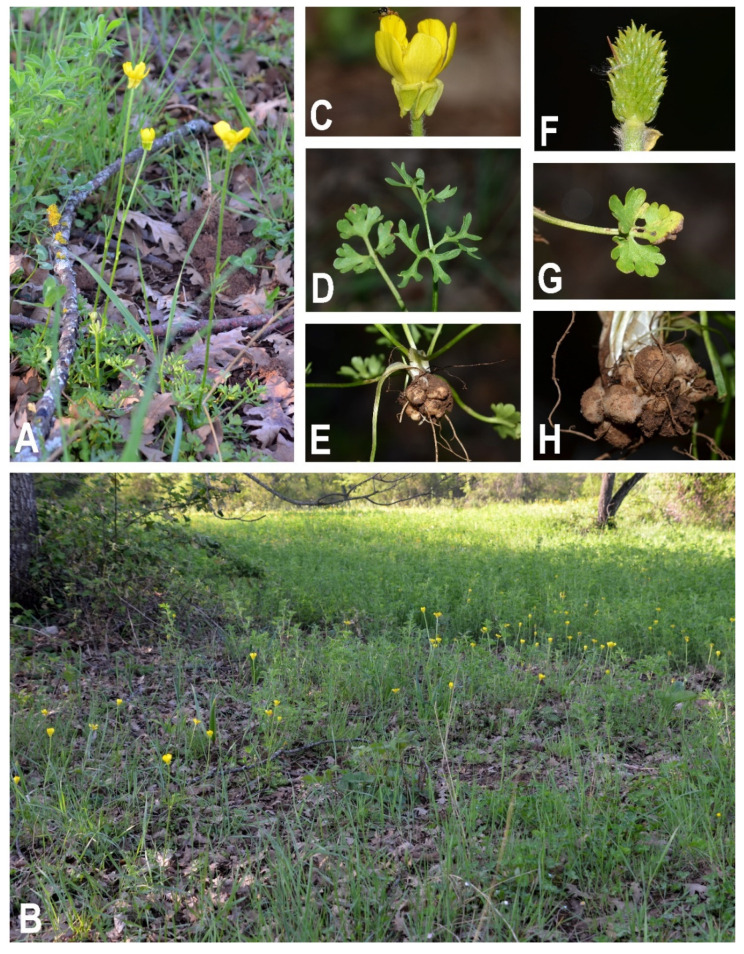
*Ranunculus gracilis* (Lazio, Mt. Campitelli, photo E. De Santis): (**A**) habit; (**B**) habitat; (**C**) flower with deflexed sepals; (**D**) inner basal leaves; (**E**) basal portion of the plant; (**F**) achenes; (**G**) outer basal leaf; (**H**) particular of the broadly ovate tubers.

Distribution: endemic to the SE Euro-Mediterranean area, distributed in Italy, Balkan Peninsula, Turkey and Georgia. The presence of *R. gracilis* in the latter country was reported by Grossheim [54], under the name *R. agerii* Bertol., for a single locality (Akhaltsikhe district; see Map No. 82 included in [54]), but, in our opinion, this report requires further checks. In Italy, in the current state of knowledge, it is present only in Lazio based on our finding reported here, no longer recorded in Emilia-Romagna and Sicily, recorded by mistake in Calabria and Puglia and formerly cultivated in botanical gardens in Toscana and Umbria (Figure 3). The presence of the species in each Italian administrative region is discussed below:

- Piemonte: the species was cited as doubtfully occurring in Piemonte by Conti et al. [42] and as recorded by mistake by Bartolucci et al. [4]; it was never recorded for the region (D. Bouvet and A. Selvaggi, pers. comm.).
- Emilia-Romagna: the presence of the species is confirmed by several old herbarium specimens kept in BOLO, CHE, FI, G, P, PI and RO (see specimens examined). Furthermore, the 16th century samples preserved in the “*En Tibi*” and “*Cibo*” herbaria were also collected in Emilia-Romagna near Bologna (Stefanaki et al. 2018, 2019). No recent herbarium samples or bibliographic records have been found (see, [55]); therefore, the species should be considered as no longer recorded. Targeted field research will be needed before considering the species as locally extinct.
- Toscana: the species was never recorded for the region. We traced an old herbarium specimen in FI, collected in the Botanical Garden of the University of Firenze, where the species was probably cultivated.
- Umbria: the species was recorded for the region in the past [9,11,15] as naturalized in the Botanical Garden of the University of Perugia. We traced the herbarium specimen linked to the old report by Fiori [11] in FI (see Specimens examined).
- Lazio: during field investigations carried out in the territory of Anagni (Frosinone, Central Italy) in March and April 2022, we discovered the species on Mt. Campitelli (Figure 4). Our finding corroborates the old report for this area by Sibilia ([56], under the name *R. agerii*) and confirms the presence of this species in Italy. The Sibilia record has never been incorporated in the regional floras [4,57,58,59]. In the current state of knowledge, this is the only population present in Italy.
- Puglia: the species was recorded by Di Pietro and Misano [60]. This record was later regarded as doubtful by Bartolucci et al. [4]. We were not able to trace herbarium specimens linked to this record and the species should be regarded as probably indicated by mistake (R. Di Pietro, pers. comm.).
- Calabria: according to Peruzzi and Passalacqua [41], the Calabrian records should be referred to *R. monspeliacus* subsp. *aspromontanus*.
- Sicily: the species was reported in Sicily from different localities by Giardina et al. [12]: between Vittoria and Terranova based on the description of *R. schowii* [18], between Catania and Misterbianco based on Strobl [61] and from Polizzi Generosa [62]. We have shown that *R. schowii* is not related to *R. gracilis;* therefore, the report of the latter between Vittoria and Terranova is erroneous. We traced, in PAL, the sample collected in Polizzi Generosa (“sotto il paese di Polizzi Generosa vicino all’acquedotto, 30 April 1990, Raimondo and Certa”), which should be referred to *R. paludosus*. A specimen cited in Wikiplantbase Sicilia [63] as *R. gracilis* and stored in PAL (No. 43515) collected at Busambra belongs to *R. paludosus* as well as a specimen in CAT No. 048272 (Monte Lauro, 9/V/1991, Brullo et al.). The only datum that we were able to confirm is the indication by Strobl [61], for the Amenano between Catania and Misterbianco at the foot of Etna, thanks to the tracing of an old herbarium sample stored in FI, collected in 1874 by Heidenreich at Misterbianco (quoted also by Fiori et al. [10]). Based on our data, *R. gracilis* should be considered as no longer recorded in Sicily. Targeted field research will be needed before considering the species as locally extinct.

Phenology: flowering late March to April; fruiting in April and May.

Habitat: humid habitats, meadows, fields and open woodland at an elevation of 0–1400 m a.s.l. In Italy, in the only currently known locality (Mt. Campitelli, Anagni, Lazio), it grows on the edge of *Quercus cerris* L. wood, on fresh and moist sandy soil, at an elevation of 750 m a.s.l.

Chromosome number: 2n = 16 [64].

Conservation status: *Ranunculus gracilis* currently occurs outside the NATURA 2000 network on Mt. Campitelli (Anagni, Frosinone) in Lazio (Central Italy). The populations in Emilia-Romagna (Northern Italy) and Sicily, confirmed by old herbarium specimens, have not been observed for over 120 years. The area of occupancy (AOO) is 4 km^2^, calculated with GeoCAT (Geospatial Conservation Assessment Tool) software [65]. The species actually occurs in one location and a decline in the AOO was observed, considering the possible extinction of some populations. According to IUCN [66] criterion B2ab(i,ii,iv), the species is assessed as Critically Endangered (CR) at the regional level (Italy).

Taxonomic remarks: *Ranuculus gracilis* belongs to *R.* sect. *Ranunculastrum*. The section is characterized by species with a beak equal to or longer than the achene body, a receptacle glabrous and with dimorphic roots partly tuberous [2]. In Italy, there are nine native taxa belonging to this section [4]: *R. garganicus* Ten., *R. illyricus* L., *R. isthmicus* Boiss., *R. millefoliatus* Vahl, *R. monspeliacus* L. subsp. *aspromontanus* (Huter, Porta & Rigo) Peruzzi & N.G.Passal. (endemic), *R. monspeliacus* L. subsp. *monspeliacus*, *R. monspeliacus* L. subsp. *saxatilis* Nyman (extinct), *R. paludosus* Poir. and *R. spicatus* Desf. subsp. *rupestris* (Guss.) Maire (endemic). *Ranunculus gracilis* is easily distinguished from other species for a specific combination of characters, such as broadly ovoid tubers, basal leaf subglabrous shallowly 3-lobed (outer), 3-partite to or near base, with lobes variously dissected into obtuse segments (inner) and sepals deflexed at flowering. In Table 1, the qualitative morphological diagnostic features [9,17,41] of the Italian native taxa belonging to *R.* sect. *Ranunculastrum* are reported.

Specimens examined of *Ranunculus gracilis* E.D.Clarke: **Italy**. EMILIA-ROMAGNA: *Reperi copiosum Bononia in campis collinis prope* i Gessi di Montedonato, April 1818, *s.coll.* (BOLO, lectotype of *R. agerii*); legi *Bononia in campis collinis di Gaibolla* inter sata, May 1823, *s. coll.* (BOLO, under the name *R. agerii*); legi Bononia a Gaibola, s.d., *s. coll.* (BOLO, under the name *R. agerii*); in collibus prope Bononiam, 27 April 1885, A. *Fiori s.n.* (CHE barcode CHE007984, P barcode P02403935, under the name *R. agerii*); *ex collibus Bononiensibus*, 1840, *M. de Martem s.n.* (P barcode P02817111, under the name *R. agerii*); environs de Bologne, Apennins, 1824, *Schleicher s.n.* (P barcode P02819312, under the name *R. agerii*); *circa Bononiam*, s.d., *s.coll.* (P barcode P06233836, under the name *R. agerii*); *legi in silvis prope Gaibola Bononiae*, April 1898, *G. Betti s.n.* (PI No. 015140); nei prati presso Gaibola, May 1884, *Mattei s.n.* (PI No. 061810, under the name *R. agerii*); colline bolognesi a M. Donato, April 1864, *s.coll.* (PI No. 061811, under the name *R. agerii*); Bologna, M. Donato, s.d., *Rosellini s.n.?* (PI No. 061812, under the name *R. agerii*); *R. agerii* Bertol., s.loc., s.d., *s.coll.* (PI No. 003289); colline bolognesi a M. Donato, April 1864, *O. Beccari s.n.* (FI barcode FI066998, under the name *R. agerii*); lungo il Ravone presso Bologna, 18 April 1894, *A. Fiori s.n.* (FI barcodes FI066999, FI067000, FI067001, under the name *R. agerii*); vallata del Ravone presso Bologna, April 1882, *Gibelli s.n.* (FI barcodes FI067002, FI067017, PI Nos 061808, 061809, under the name *R. agerii*); Gaibola presso Bologna, nei prati, April 1891, *Mattei s.n.* (FI barcode FI FI067003, under the name *R. agerii*); Bologna, June 1889, *Mattei s.n.* (FI barcode FI067004, under the name *R. agerii*); vicinanze di Bologna, June 1883, *Mattei s.n.* (FI barcode FI067005, under the name R. agerii); prati presso Gaibola nelle vicinanze di Bologna, May 1886, *Mattei s.n.* (FI barcode FI067006, under the name *R. agerii*); nei campi alla Croara, April 1873, *G. Cugini s.n.* (FI barcode FI067007, under the name *R. agerii*); Bologna, Monte S. Donato, 25 May 1873, *G. Bertoloni s.n.* (FI barcode FI067008, under the name *R. agerii*); in pratis Gaibola prope Bononiam, 27 April 1886, A. Fiori s.n. (FI barcode FI067009, under the name *R. agerii*); nei colli Bolognesi, s.d., *G. Bertoloni s.n.* (FI barcode FI067010, under the name *R. agerii*); prati e vigne presso Gaibola Bologna, April 1885, *A. Baldacci s.n.* (FI barcode FI067011, under the name *R. agerii*); *ex collibus Bononiensibus* a Montedonato, 1842, *A. Bertoloni s.n.* (FI barcode FI067012, under the name *R. agerii*); *legi in parte meridionale montis* Castello della Croara (in cultis dia 4 May 1837), *s.coll.* (FI barcode FI067013, under the name *R. agerii*); contorni di Bologna, 1834, *Bubani s.n.* (FI barcode FI067014, under the name *R. agerii*); nei colli Bolognesi, s.d., *s.coll.* (FI barcode FI067018, under the name *R. agerii*); lungo il Ravone presso Bologna, 18 April 1892, *A. Fiori s.n.* (FI barcode FI067022, under the name *R. agerii*); Bologna, April 1892, *A. Fiori s.n.* (FI barcode FI067000, under the name *R. agerii*); Bononiae, s.d., *Moricand s.n.* (G barcode G00145280, under the name *R. agerii*); Monte Donato presso Bologna, s.d., *Bertoloni s.n.* (RO, under the name *R. agerii*); Bologna, May 1839, *s.coll.* (RO, under the name *R. agerii*); Barbianello, Bolognese, terreno argilloso, May 1899, *Betti s.n.* (RO, under the name *R. agerii*); Rio Ravone presso Bologna, 27 April 1885, *A. Fiori s.n.* (RO, under the name *R. agerii*); in un campo presso Pontecchio e nei prati a Gaibola, pre. di Bologna, April-May 1882, *G. Pirzini s.n.* (RO); TOSCANA. Florentia, 14 April 1866 (ex horto botanico Musei) (FI barcode FI067019, under the name *R. agerii*); UMBRIA. Perugia: largamente inselvatichito nell’Orto Botanico dell’Università, ma proveniente dal Bologense, 6 April 1899, *L. Palomba s.n.* (FI barcode FI067020, under the name *R. agerii*); LAZIO. Monti Campitelli (Anagni, Frosinone), radure e margine boschivo, 730 m, 20 April 2022, *E. De Santis s.n.* (APP No. 66136); SICILIA. Catania, in humidis pr. Misterbianco, 24 March 1874, *Heindereich s.n.* (FI barcode FI067021, under the name *R. agerii*). **Greece.** Samaria (Criti), 25 April 1976, *W. Greuter s.n.* (P barcode P00040497); Bassoré (Grèce), 1200 m, 23 April 1986, *F. Billy s.n.* (CLF barcode CLF006633); Montes Argolidis, s.d., *Boissier s.n.* (JE barcode JE 00021604) L; Insel Lefkádha (Nom. Lefkádhos). Kolliváta: Vorhof und Umgebung der Friedhofs östlich oberhalb der Ortschaft (ca. 1 km E Aléxandhros) (UTM 34S DH 7388), frische (wechseltrockene) Rudertalfluren, 445 m, 24 April 2011, *Gutermann W.* et al. *Iter Ionicum XIX 39880* (WU barcode WU0085740); Greece. Cephalonia. Grizata (Γριζάτα). Surrounding of gorge approx. 600 m NW of Grizata church, Maquis, (MGRS 34S DH 6830), 60–90 m, 30 March 2005, *Gilli C.* et al. *Iter Ionicum XXI 41217* (WU barcode WU0091948); Insel Kérkira (Nom. Kerkíras). Pandokrátoras: Karstplateau der Westseite, bei den großen Dolinen südlich des Fahrwegs (0.7–1.2 km westlich des Gipfels). [UTM: 34S DK 0200], 750–770 m, Rasenfragmente der Dolinen-Sonnseite, 16 May 2000, *Gutermann, W.* et al. *Iter ionicum XVIII 34982* (WU barcode WU 0097318); Griechenland: Jonische Inseln: Kefallinia. Umgebung von Sámi: Gebiet zwischen der Stadt Sámi und der Ortschaft (Halbinsel) Dhihália sowie der Moní Iperajías Theotóku Agrilíon (ca. 2 km NE oberhalb von Sámi), 0–ca. 200 m, 11 April 1974, *Fischer M.A. & Fischer G. s.n.* (WU barcode WU0097319); Corfù, Monte Deca, 25 April 1887, *Gelmi s.n.* (WU barcode WU0097320); Corcyra (Corfu) Im Gerölle am Mte unter Büschen von Quercus coccifera, 11 April 1877, *Spreitzenhofer G.C. Iter jonicum a. 1877 s.n*. (WU No. 0097321); Corcyra (Corfu), am Plateau des Mte. San Salvatore, 13 April 1877, *Spreitzenhofer G.C. Iter jonicum a. 1877 127* (WU No. 0097322); Corcyra (Corfu) Mte. Deca im Gerölle in circa 15-1600 Fuß Höhe, in der Richtung gegen San Deca, 11 April 1877, *Spreitzenhofer G.C. Iter jonicum a. 1877 62* (WU No. 0097323); Zakynthos. Kulturland bei der Weggabelung (am Fahrweg zum Vrachiónas) 1 km NE Mariés (UTM 34S DG 7285) ca. 460 m, Flach terrassierte, steinige Olivenhaine, angrenzende Cistus-Heide, 25 April 2012, *Gutermann W.* et al. *Iter Ionicum XX 40612* (WU No. 0102503); Zante, Nördl. von Volimäs, 26 March 1936, *K. Ronniger s.n.* (W qrcode W0148865); S. Deka; prope ecclesiam Pantokrator, 529 m, 13 April 1896, Baenitz C. s.n. (DR barcode DR057363, under the name *R. agerii*; mixed with *R. paludosus*); colles elati Argolidis et Arkadiae, Apr. 1842, *Boissier s.n.* (MW barcode MW0592427); **Turkey**. Sinop, Ince Burum at Gerne, 1 May 1967, *Tobey C. 1616A* (E barcode E00442245, E00442247); Sinop, Ince Burum, 30 m, from clearings in woods, moist, 23 April 1966, *Tobey C. 1616A* (E barcode E00442245); *ibidem*, volcanicconglomerate, seaside. Also in forest clearings, 23 April 1966, *Tobey C. 1616* (E barcode E00442244); Bolu, between Istanbul and Ankara, E. of Gerede, short grassy turf, with Pinus, Juniperus and Quercus scrub, 28 April 1970, *Rix E. M. 1514* (E barcode E00442246); Istanbul, Silivri: Near Beyciler, edge of shrubby community, dry meadow, 16 April 1961, *Demiriz H. 4388* (E barcode E00442249); Istanbul (As.), Yakacik-Aydos road, Fountain, maquis, 6 May 1946, *Demiriz H. 2498* (E barcode E00442250); Aziatisch Turkije, Zonguldak (a.d. Zwarte Zee), 1 May 1947, Dijkstra, SJ s.n. (L qrcode L.1745470); Istanbul, Proti, 23 April 1938, *Post B.v.D. s.n.* (E barcode E00442253); Istanbul, Camlica, 26 April 1919, *Post B.v.D. s.n.* (E barcode E00442405); Istanbul, Cypress Hill grave, 25 April 1918, *Post B.v.D. s.n.* (E barcode E00442243); Bithynia, 1839, *Grisebach s.n.* (BR barcode BR0000005295913); in m. Bulgurlu, *Grisebach 24* (GOET barcode GOET009797).

## 4. Conclusions

Nomenclature plays a central role in the description of the diversity of life on our planet and the typification process is essential for any taxonomic study. At the same time, floristic research and the study of herbarium collections are of crucial importance in biodiversity conservation of vascular plants and are necessary to collect data for planning the correct conservation strategies. Our study on *R. gracilis* in Italy allowed us, primarily, to evaluate the taxonomic identity of *R. agerii* and *R. schowii*, both described from Italian territory. After typification, *R. agerii* should be regarded as a heterotypic synonym of *R. gracilis*, while *R. schowii* showed a combination of unique characters, close in some ways to atypical forms of *R. isthmicus*, and needs further studies to assess the morphological variability in the species and to clarify its taxonomic status. Thanks to our contribution, the presence of *R. gracilis* in Italy is confirmed, expanding the distribution range of this endemic species to the SE Euro-Mediterranean towards the west. In Italy, *R. gracilis* is present, in the current state of knowledge, with a single population at risk of extinction found in Lazio (Central Italy). Furthermore, we were able to confirm the historical presence of the species, based on the study of herbarium collections, in the Emilia-Romagna (Northern Italy) and Sicily, where it has not been observed for over 120 years. It will now be possible to plan specific field surveys to verify whether *R. gracilis* is still present in these areas or is to be considered extinct. Furthermore, the species is now reported in the national conservation framework.

## Figures and Tables

**Table 1:** Comparisons of morphological qualitative characters among native species of *R.* sect. *Ranunculastrum* in Italy.

	*R. gracilis*	*R. garganicus*	*R. illyricus*	*R. isthmicus*	*R. millefoliatus*	*R. monspeliacus* subsp. *monspeliacus*	*R. monspeliacus* subsp. *aspromontanus*	*R. monspeliacus* subsp. *saxatilis*	*R. paludosus*	*R. spicatus* subsp. *rupestris*
Basal leaf shape	3-lobed to 3-partite divided into obtuse, ovate or oblong lobes (outer), 3-partite to or near base, with lobes variously dissected into linear, obtuse segments (inner)	3-partite to base, lobes 2–3 pinnatisect into linear-lanceolate subacute segments	3-partite to base into linear-lanceolate lobes, entire or tripartite, rarely simply pinnatisect	3-partite to base or pinnatisect with lobes dissected in broadly linear, obtuse segments	3-partite, lobes 2–3 pinnatisect into linear-lanceolate subacute segments	3-partite to base, with lobes 3-fid (the middle stipitate)	orbicular to3-lobed	orbicular to3-lobed	flabellate to sub-orbicular (outer), 3-partite (inner) to base and usually divided into broadly linear segments	orbicular to 3-lobed
Basal leaf indumentum	glabrous to subglabrous	subglabrous	lanate-subsericeous	sparsely appressed-pubescent	glabrous	pubescent	pubescent	sericeous	appressed pubescent to subglabrous	appressed pubescent
Tubers shape	broadly ovoid	cylindrical	ellipsoid to broadly fusiform	fusiform	ovoid	fusiform	fusiform	fusiform	ellipsoid or broadly cylindrical	fusiform
Sepals	deflexed at flowering	appressed to corolla at flowering	deflexed at flowering	deflexed at flowering	appressed to corolla at flowering	deflexed at flowering	deflexed at flowering	deflexed at flowering	appressed to corolla at flowering	appressed to corolla at flowering
Achene indumentum	glabrous	glabrous	glabrous	glabrous	glabrous	pubescent	pubescent	pubescent	glabrous or pubescent	pubescent

## Data Availability

The data presented in the current study are available within the article.
